# Genomic Alterations Identification and Resistance Mechanisms Exploration of NSCLC With Central Nervous System Metastases Using Liquid Biopsy of Cerebrospinal Fluid: A Real-World Study

**DOI:** 10.3389/fonc.2022.889591

**Published:** 2022-06-23

**Authors:** Fangfang Shen, Naixin Liang, Zaiwen Fan, Min Zhao, Jing Kang, Xifang Wang, Qun Hu, Yongping Mu, Kai Wang, Mingming Yuan, Rongrong Chen, Wei Guo, Guilan Dong, Jun Zhao, Jun Bai

**Affiliations:** ^1^ Department of Respiratory Medicine, Shanxi Province Cancer Hospital, Shanxi Hospital Affiliated to Cancer Hospital, Chinese Academy of Medical Sciences, Cancer Hospital Affiliated to Shanxi Medical University, Taiyuan, China; ^2^ Department of Thoracic Surgery, Peking Union Medical College Hospital, Peking Union Medical College and Chinese Academy of Medical Sciences, Beijing, China; ^3^ Department of Medical Oncology, Air Force Medical Center, Chinese People's Liberation Army (PLA), Beijing, China; ^4^ Department of Oncology, Hebei Chest Hospital, Research Center of Hebei Lung Cancer Prevention and Treatment, Shijiazhuang, China; ^5^ Department of Oncology, Honghui Hospital, Xi’an Jiaotong University, Xi’an, China; ^6^ Department of Medical Oncology, Shaanxi Provincial People’s Hospital, Xi’an, China; ^7^ Department of Oncology, The Affiliated Hospital of Inner Mongolia Medical University, Hohhot, China; ^8^ Department of Clinical Laboratory Center, The Affiliated People’s Hospital of Inner Mongolia Medical University, Inner Mongolia Autonomous Region Cancer Hospital, Hohhot, China; ^9^ Medical Center, Geneplus-Beijing, Beijing, China; ^10^ Department of Medical Oncology, Tangshan People’s Hospital, Tangshan, China; ^11^ Key Laboratory of Carcinogenesis and Translational Research (Ministry of Education/Beijing), Department I of Thoracic Oncology, Peking University Cancer Hospital and Institute, Beijing, China

**Keywords:** cerebrospinal fluid, resistance mutations, real-world study, non-small cell lung cancer, central nervous system metastases

## Abstract

**Background:**

Genomic profiling of cerebrospinal fluid (CSF) can be used to detect actionable mutations and guide clinical treatment of non-small cell lung cancer (NSCLC) patients with central nervous system (CNS) metastases. Examining the performance of CSF samples in real-world settings can confirm the potential of CSF genotyping for guiding therapy in clinical practice.

**Patients and Methods:**

We included 1,396 samples from 970 NSCLC patients with CNS metastases in real-world settings. All samples underwent targeted next-generation sequencing of 1,021 cancer-relevant genes. In total, 100 CSF samples from 77 patients who had previously received targeted treatment were retrospectively analyzed to explore the mechanisms of TKI-resistance.

**Results:**

For NSCLC patients with CNS metastases, CSF samples were slightly more often used for genomic sequencing in treated patients with only distant CNS metastases compared to other patients (10.96% vs. 0.81–9.61%). Alteration rates in CSF samples were significantly higher than those in plasma, especially for copy number variants (CNV). The MSAFs of CSF samples were significantly higher than those of plasma and tumor tissues (all p <0.001). Remarkably, detection rates of all actionable mutations and *EGFR* in CSF were higher than those in plasma samples of treated patients (all p <0.0001). For concordance between paired CSF and plasma samples that were simultaneously tested, the MSAF of the CSF was significantly higher than that of matched plasma cfDNA (p <0.001). From multiple comparisons, it can be seen that CSF better detects alterations compared to plasma, especially CNV and structural variant (SV) alterations. CSF cfDNA in identifying mutations can confer the reason for the limited efficacy of EGFR-TKIs for 56 patients (78.87%, 56/71).

**Conclusions:**

This real-world large cohort study confirmed that CSF had higher sensitivity than plasma in identifying actionable mutations and showed high potential in exploring underlying resistance mechanisms. CSF can be used in genomics profiling to facilitate the broad exploration of potential resistance mechanisms for NSCLC patients with CNS metastases.

## Introduction

Lung cancer, one of the most common cancers, remains the most common cause of cancer-related deaths, with high global morbidity and mortality (1). Non-small cell lung cancer (NSCLC), the most frequent (85–90%) cause of malignant lung cancers (2), is also the most common source of central nervous system (CNS) metastases (3). CNS metastases are a frequent and severe complication associated with NSCLC, which occurs in 20–25% of advanced NSCLC patients at initial presentation and is seen in 30–40% of NSCLC patients during their disease (4–6). The median and 1-year overall survival for patients with brain metastases is only 3–7 months and 29.9%, respectively (5–7), and the treatment options for NSCLC with CNS metastases are limited, with most current clinical trials, excluding them. Currently, treatment of NSCLC with CNS metastases is multidisciplinary and involves chemotherapy, radiation therapy, and targeted therapy (5, 8–10).

Next-generation sequencing (NGS)-based genetic testing, which provides abundant genetic information about cancer, has developed therapeutic strategies against driver mutations such as epidermal growth factor receptor (*EGFR*), anaplastic lymphoma kinase (*ALK*), and ROS proto-oncogene 1 (*ROS1*), for NSCLC (11). NGS may also indicate whether NSCLC patients can be treated with immunotherapy. Immunotherapy-based treatments are of greater benefit to non-oncogene addicted NSCLC patients and significantly less effective in the EGFR population (12, 13). In NSCLC with CNS metastases, CNS metastases have distinct genetic information. Thus, performing intracranial biopsy and genetic testing for molecular information and acquired resistance is pertinent (14). Owing to invasive and time-consuming procedures when accessing CNS metastases and the heterogeneity between intracranial and extra-cranial lesions (15, 16), it becomes difficult for NSCLC patients with CNS metastases to access information on genetics or resistance mechanisms. Thus, an urgent need to discover specimens for genetic testing in NSCLC patients with CNS metastases exists.

Liquid biopsy using circulating tumor DNA (ctDNA), which is used in various body effluents, namely, blood, cerebrospinal fluid, ascitic fluid, pleural fluid, and urine instead of tumor tissue, has been widely used in clinical practice to detect genomic alterations in NSCLC (17–19). It can non-invasively identify actionable alterations and overcome both spatial and temporal tumor heterogeneity not addressed by tissue biopsy (17). Liquid biopsy using plasma ctDNA has been widely used in clinical practice, and many studies have demonstrated its feasibility. The new International Association for the Study of Lung Cancer (IASLC) liquid biopsy consensus statement in 2021 noted that liquid biopsy was the preferred method of molecular testing in some clinical settings and proved complementary to tumor tissue testing in others (20). However, owing to the blood–brain barrier, the sensitivity of plasma ctDNA sequencing is limited in NSCLC patients with CNS metastases (21). The 2021 IASLC liquid biopsy statement suggested CSF as an emerging alternative ctDNA source for detecting gene alterations and clonal heterogeneity in patients with CNS metastases (20). Previous research also suggested that cerebrospinal fluid (CSF)-cell free DNA (cfDNA) could reveal unique genetic profiles of intracranial metastases and guide clinical treatment of NSCLC patients with CNS metastases (21–24). However, the potential use of CSF as a liquid biopsy source remains to be examined in a real-world setting.

To provide more implications for the clinical application of liquid biopsy using CSF and explore its potential to identify actionable mutations and explore underlying resistance mechanisms for NSCLC patients with CNS metastases, we analyzed 1,396 samples from 970 NSCLC patients with CNS metastases (brain metastases [BM] and leptomeningeal metastases [LM]) who underwent NGS in real-world settings.

## Material and Methods

### Clinical Cohort

In this retrospective cohort study, a cohort of 970 NSCLC patients with CNS metastases (BM and LM) was enrolled at the Geneplus Medical Laboratory (Beijing, China) from May 2019 to July 2021. The diagnosis criteria for BM were based on metastatic lesions detected on brain magnetic resonance imaging, while the diagnostic criteria for LM were based on tumor cells detected in CSF samples or leptomeningeal enhancement on brain magnetic resonance imaging. To analyze the real-world efficacy of CSF in detecting actionable mutations, all patients underwent NGS in a laboratory accredited by the College of American Pathologists. Demographic, clinicopathological, and tumor histopathological results, such as TNM stag, metastatic sites, and cellular differentiation grade, were obtained for each patient. This study was approved by the Institutional Review Board of Shaanxi Provincial People’s Hospital. All subjects provided written informed consent before undergoing any study-related procedures. This study was conducted in accordance with the principles of the Declaration of Helsinki.

### Sample Processing and DNA Extraction

Within 72 h of collection, peripheral blood samples were centrifuged to obtain plasma and white blood cells (WBCs). The CSF supernatant was centrifuged to separate it from the cell sediment. All tissue samples, including fresh and formalin-fixed paraffin-embedded (FFPE) tissue samples, underwent pathological assessment to confirm histologic classification and adequacy of the tumor tissues, which required a minimum of 20% tumor content. Circulating cell-free DNA (cfDNA) was isolated from the CSF supernatant and plasma using a QIAamp Circulating Nucleic Acid Kit (Qiagen, Hilden, Germany). Genomic DNA (gDNA) from WBCs and tumor tissues was extracted using the DNeasy Blood & Tissue Kit (Qiagen, Hilden, Germany). Circulating cfDNA from other body fluids were processed into indexed libraries, as discussed in previous studies (25–28).

### Library Preparation and Target Enrichment

DNA concentration was measured using the Qubit fluorometer (Invitrogen, Carlsbad, CA, USA) and the Qubit dsDNA HS (High Sensitivity) Assay Kit (Invitrogen, Carlsbad, CA, USA). The size distribution of circulating cfDNA was assessed using the Agilent 2100 BioAnalyzer and DNA HS kit (Agilent Technologies, Santa Clara, CA, USA). The SeqCap EZ Library system (Roche NimbleGen, Madison, WI, USA) was used for target enrichment. In total, 1,386 libraries from 970 patients were hybridized to custom-designed biotinylated oligonucleotide probes (IDT, Coralville, IA, USA) covering 1.6 Mbp of the genome, and the captured genomic regions included 1,021 cancer-related genes (**Table S1**). The captured DNA fragments were amplified after hybrid selection and then pooled into several multiplexed libraries. Sequencing was performed using the Illumina Nextseq CN 500 (Illumina, San Diego, CA, USA) or the Gene^+^Seq-2000 Sequencing System (GenePlus-Suzhou, Suzhou, China), according to the instructions of the manufacturer.

### Sequencing and Data Analysis

Sequencing data were analyzed using default parameters. After the removal of terminal adaptor sequences and low-quality reads, the clean reads were aligned to the reference human genome (hg19) using the Burrows–Wheel Aligner (BWA; version 0.7.12-r1039) with default parameters. Base quality recalibration and local realignment were performed using the Gene Analysis Toolkit (GATK; version 3.4-46-gbc02625). Somatic single nucleotide variations (SNVs) and insertion or deletion of small fragments (Indels) were determined by the MuTect2 algorithm. The Contra algorithm (version 2.0.8) was used to detect somatic copy number alterations. All candidate fusion genes were manually mapped to the initial cfDNA fragments using unique barcoding and alignment information. The minimal mean effective depth was 300×, 1000×, and 1,000× in tissue, CSF, and plasma samples, respectively.

### Statistical Analysis

Associations between two or more categorical variables were analyzed using Fisher’s exact or Chi-square tests. The comparison of means among three or more groups was performed using one-way ANOVA tests. All statistical analyses and presentations were performed using R v3.6.3. All tests were two-sided, and *p*-values <0.05 represented statistical significance.

## Results

### Study Design and Patient Demographics

In total, 970 patients (49.90%, male) with stage IV NSCLC and CNS metastases were enrolled in this study. Patient characteristics are presented in **Table 1**. Among patients, adenocarcinoma had the largest proportion (82.27%), followed by squamous carcinoma (2.99%), adenosquamous carcinoma (0.72%), and large cell carcinoma (0.21%). Cellular morphology was unidentified in 134 (13.81%) NSCLC cases. The median age at diagnosis was 57 (range, 18–91) years. Most patients (962/970) were diagnosed with BM, 16 had LM, and eight had both BM and LM.

**Table 1:** Patient Characteristics.

Characteristic	Number	Percentage (%)
**Age (years)**		
Median	57	–
Range	18–91	–
**Gender**		
Male	484	49.90
Female	486	50.10
**Histology subtype**		
Adenocarcinoma	798	82.27
Squamous	29	2.99
Adenosquamous	7	0.72
Large cell	2	0.21
NA	134	13.81
**Specimen**	N = 1,396*	
Cerebrospinal fluid	119	8.52
Tumor tissue	416	29.80
Plasma	791	56.66
Other**	70	5.01
Previous treatment		
No	240	17.19
Yes	1,156	82.81

*Including 282 patients had multiple samples tested simultaneously or consequently. **Including 59 pleural effusion samples, 5 already extracted DNA, 4 pericardial fluid samples, one brushing washing fluid, and one ascites. NA, not available.

**Figure 1:**
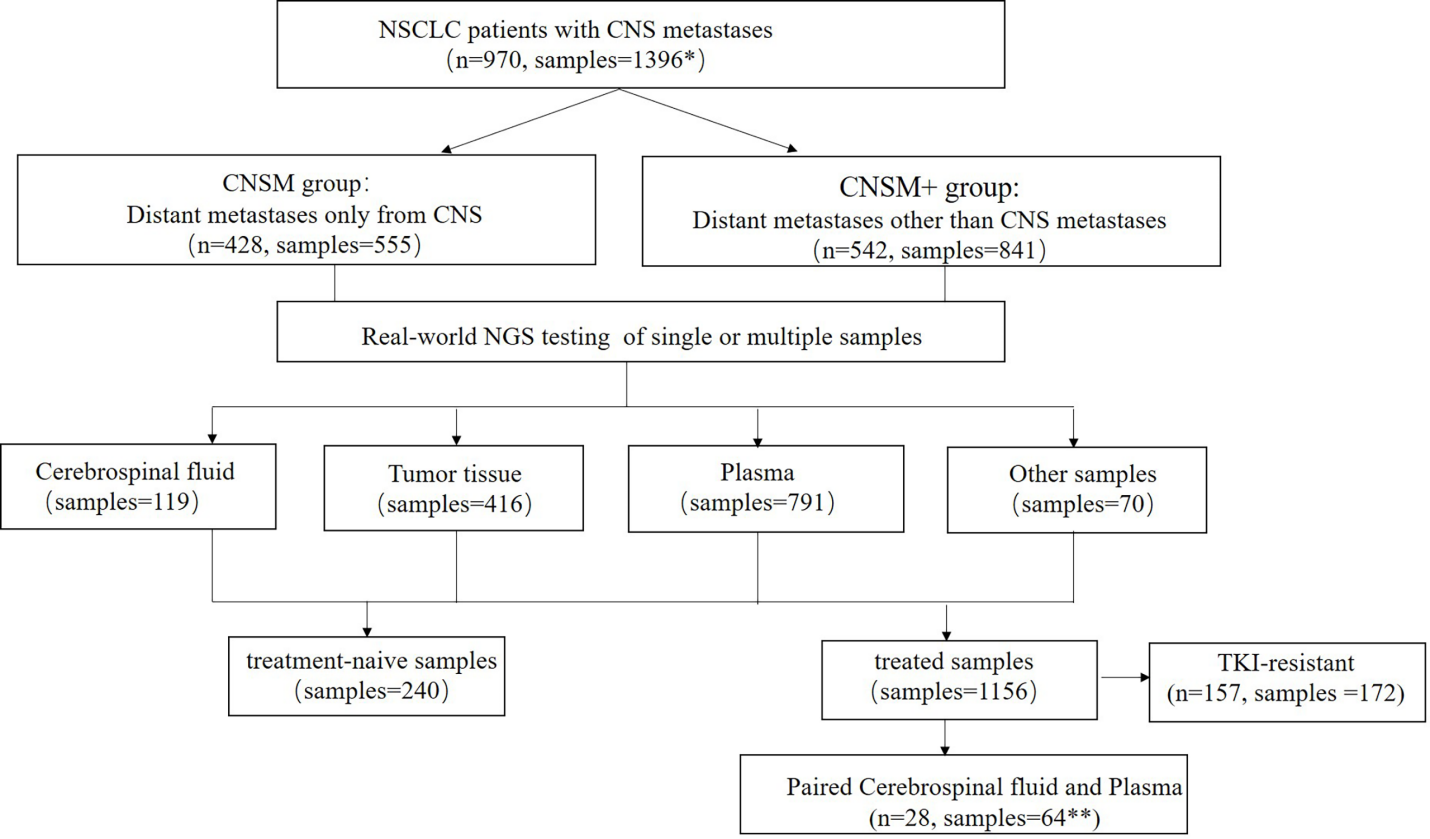
Study design. NSCLC, non-small cell lung cancer; CNS, central nervous system; NGS, Next-generation sequencing. *Among the 970 patients in this study, 688 patients had one sample tested, while 282 patients had multiple samples tested simultaneously or consequently. **Three patients had two or more paired cerebrospinal fluid and plasma samples. CNSM group, patients who had distant metastases only from the CNS, CNSM+ group, patients with other organ involvement, and distant metastases other than CNS metastases.

In this real-world setting, 119 CSF, 416 tumor tissue, 791 plasma, and 70 other samples were collected. The 416 tissue samples included 269 primary tissues, 46 intracranial metastatic tissues, 39 lymph node tissues, 26 other metastatic tissues, and 36 tissues of unknown origin, while the 70 other samples included 59 pleural effusion samples, 5 already extracted DNA, 4 pericardial fluid samples, one brushing washing fluid, and one ascitic fluid. Most patients (86.7%, 688/970) had a single CSF, tissue, plasma, other body fluid sample, or DNA tested, while 282 patients had multiple samples tested simultaneously or consequently. Meanwhile, 240 samples were treatment-naive, containing 2 (0.83%) CSF, 129 (53.75%) tissue, 98 (40.83%) plasma, and 11 (4.58%) other samples; and 1,156 were treated, comprising 117 (10.12%) CSF, 287 (24.83%) tissue, 693 (59.95%) plasma, and 59 (5.10%) other samples (**Figure 1**).

Among 970 patients included in this study, 428 (44.12%) had distant metastases only from the CNS (CNSM group), and 542 (55.88%) patients had other organ involvement and distant metastases other than CNS metastases (CNSM+ group). Based on whether they were treated samples or distant metastases other than CNS metastases, the 1,396 samples from these 970 NSCLC patients with CNS metastases were divided into four groups: the treatment-naive CNSM group (n = 117), the treatment-naive CNSM+ group (n = 123), the treated CNSM group (n = 438), and the treated CNSM+ group (n = 718).

**Figure 2:**
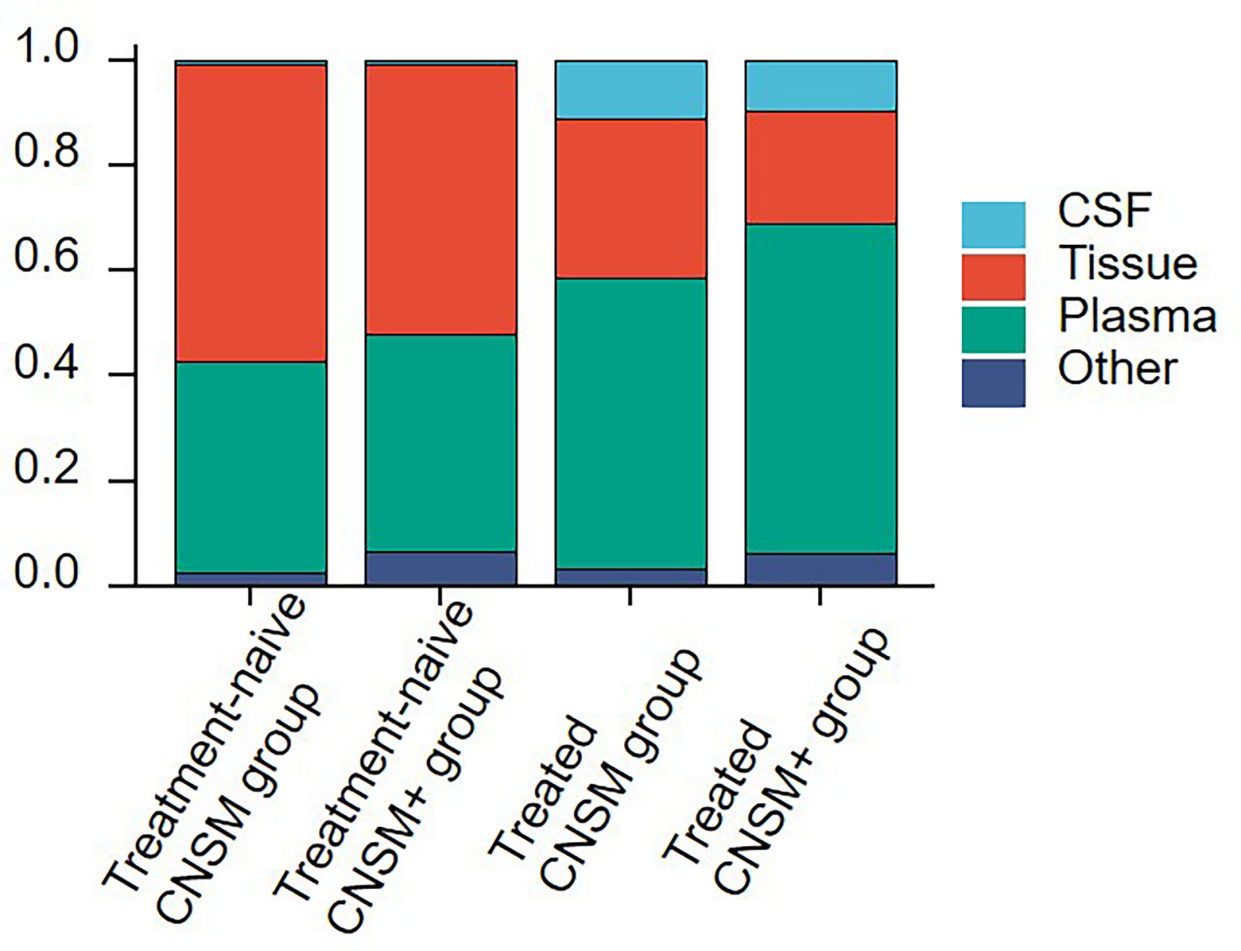
Sample selection for genetic profiling in NSCLC patients with CNS metastases. The 1,396 samples were divided into the following 4 groups according to treatment history and metastasis sites: the treatment-naive CNSM group (n = 117), the treatment-naive CNSM+ group (n = 123), the treated CNSM group (n = 438), and the treated CNSM+ group (n = 718). CNSM group, patients had distant metastases only from the CNS, CNSM+ group, patients with other organ involvement and distant metastases other than CNS metastases.

Comparisons between the four groups revealed the choice of specimen type for genetic profiling in NSCLC patients with CNS metastases in real-world settings (**Figure 2**). Among treatment-naive patients, tissue samples (51.22–56.41%) were the most examined, followed by plasma (40.17–41.46%), other samples (2.56–6.50%), and CSF (0.81–0.85%). Among treated patients, plasma samples (55.02–62.95%) were the most examined, followed by tissue (21.31–30.59%), CSF (9.61–10.96%), and other samples (3.42–6.13%), regardless of other organ involvement (**Figure 2**).

Moreover, plasma samples were slightly more often used in the treated CNSM+ group than in other groups (62.95% vs. 40.17–55.02%). And the treated CNSM group (10.96%) had the largest proportion of CSF samples (**Figure 2**).

### CSF in Real-World Setting

To further analyze the efficacy of CSF samples in real-world settings, 970 NSCLC patients with CNS metastases were retrospectively analyzed. All samples were subjected to targeted NGS of 1,021 cancer-relevant genes. **Table S2** provides a detailed list of somatic alterations identified in each patient sample.

**Figure 3:**
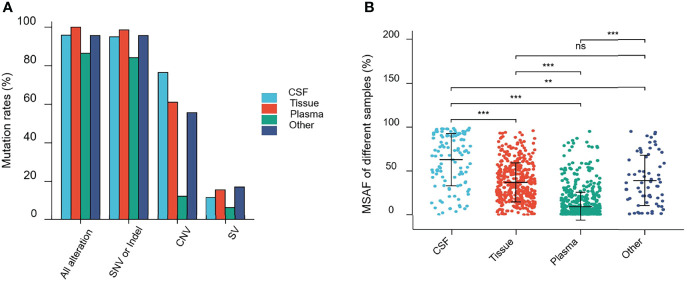
Mutations rates and Maximal somatic allele frequency (MSAF). **(A)** Mutations detected in different samples from patients in this study. **(B)** The MSAFs of CSF, tissue, plasma, and other samples from patients in this study. ns, p ≥0.05; **p <0.01; ***p <0.001.

Alterations were identified in 114 (95.80%) CSF samples and 416 (100%) tumor tissue samples, compared to 684 (86.47%) plasma and 67 (95.71%) other samples. The detection rate of all alterations in CSF was lower than that in tumor tissues (95.80% vs. 100%, p <0.001) but higher than that in the plasma (95.80% vs. 86.47%, p <0.001) (**Figure 3A**).

As shown in **Figure 3A**, 94.96% (113/119), 98.56% (410/416), 84.20% (666/791), and 95.71% (67/69) of CSF, tumor tissue, plasma cfDNA, and other samples, respectively, showed detectable SNV or Indel somatic alterations. The copy number variant (CNV) and structural variant (SV) alteration rates in different samples were also compared. The detection rate of SNVs or Indels in CSF samples was lower than that in tumor tissues (94.96% vs. 98.56%, p = 0.0193). However, the CNV alteration rate in CSF samples was significantly higher than that in tumor tissues (76.47% vs. 61.06%, p = 0.0019), and SV detection rate was not statistically different between CSF and tumor tissues (11.76% vs. 15.63%, p = 0.2952). Furthermore, the SNV or Indel, CNV, and SV alteration rates in CSF samples were significantly higher than that in plasma (SNV or Indel, 94.96% vs. 84.20%, p <0.001; CNV, 76.47% vs. 12.39%, p <0.0001; SV, 11.76% vs. 6.57%, p <0.05). This indicated that compared to plasma, CSF exhibited more CNV alterations (**Figure 3A**).

The maximal somatic allele frequency (MSAF) of the CSF was compared to that of other types of samples (**Figure 3B**). The MSAFs of CSF samples were significantly higher than those of plasma and tumor tissues, with the MSAFs of tumor tissues being significantly higher than those of plasma (all p <0.001).

**Figure 4:**
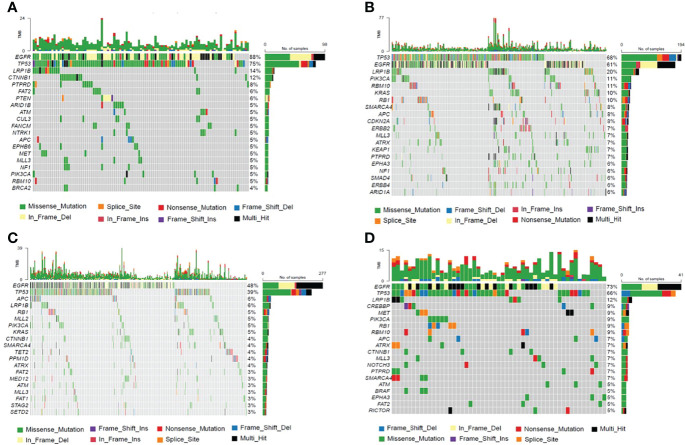
The Frequency Spectrum for treated patients with detectable SNV or Indel mutations. **(A)** Mutation frequency spectrum in treated-CSF samples (six samples were not detected; they only showed the twenty most frequently mutated genes). **(B)** Mutation frequency spectrum in treated-tumor tissue samples (three samples were not detected; they only showed the twenty most frequently mutated genes). **(C)** Mutation frequency spectrum in treated-plasma (117 samples were not detected; they only showed the twenty most frequently mutated genes). **(D)** Mutation frequency spectrum in treated-other samples (two samples were not detected; they only showed the twenty most frequently mutated genes).

Genetic profiles of CSF cfDNA, tumor tissue, plasma, and other body fluid samples or DNA from treated patients were compared. In treated-CSF samples, most recurrent mutations were observed in the *EGFR* gene, followed by *TP53*, which is the same as in treated plasma and other samples (other body fluid samples or DNA) types. Among treated-tumor tissues, the most recurrent mutations were observed in *TP53*, followed by *EGFR* (**Figure 4**), which indicated that *EGFR* and *TP53* were the two most frequently mutated genes in all sample types, as previously reported (16).

We performed a subgroup analysis, to more accurately analyze the detectability of different fluid biopsies (CSF and plasma) in patients with different metastatic sites in real-world settings. Due to the small number of treatment-naive patients who opted for CSF testing, this analysis was performed on treated patients. On the basis of distant metastases other than the CNS metastases, samples were divided into two groups as follows: distant metastases only from the CNS group (CNSM group) and distant metastases other than CNS metastases group (CNSM+ group). We analyzed mutation rates and MSAF of CSF and plasma in the two groups of treated patients.

**Figure 5:**
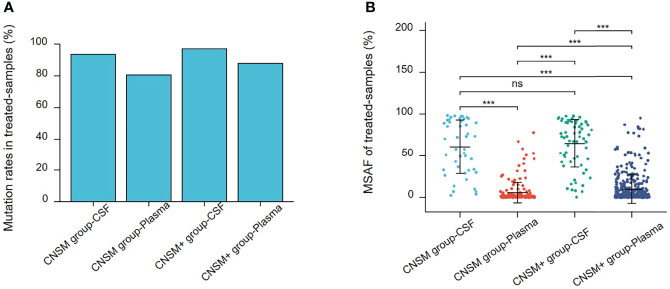
Mutations rates and MSAF of treated samples. **(A)** Mutations detected in different treated samples from patients in the treated CNSM or CNSM+ groups. **(B)** MSAFs of CSF and plasma samples from patients in the treated CNSM or CNSM+ groups. ns, p ≥ 0.05; ***p < 0.001. CNSM group, patients had distant metastases only from the CNS, CNSM+ group, patients with other organ involvement and distant metastases other than CNS metastases.

The mutation rate in CSF samples was significantly higher than that in the plasma in both the treated CNSM and CNSM+ groups (CNSM group, 93.75% vs. 80.50, p = 0.0121; CNSM+ group, 97.10% vs. 88.05%, p = 0.0066) (**Figure 5A**). For plasma samples, the mutation rate in the treated CNSM+ group was significantly higher than that in the treated CNSM group (88.05% vs. 80.50, p = 0.0003) (**Figure 5A**). There was no significant difference in the mutation rate between the treated CNSM and CNSM+ groups (93.75% vs. 97.10%, p = 0.3756) (**Figure 5A**). For MSAF of treated-samples, MSAFs of CSF samples were significantly higher than those of plasma in both the CNSM and CNSM+ groups (all p <0.001), and MSAFs of plasma in the CNSM+ group were significantly higher than those of the CNSM group (p <0.001) (**Figure 5B**).

### Driver Gene Alterations in CSF

**Figure 6:**
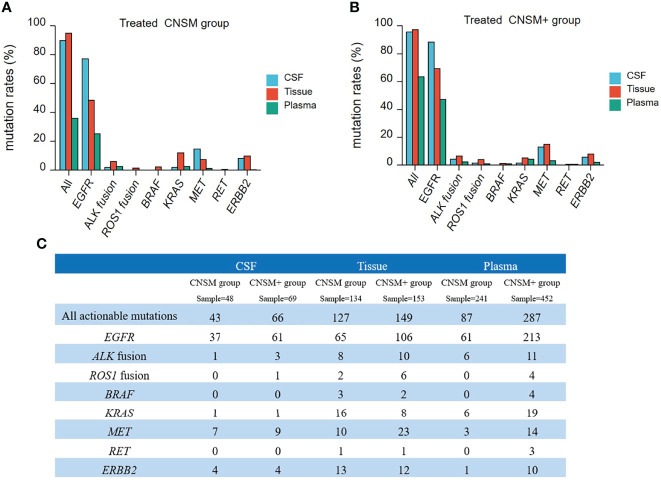
Actionable mutations rates in treated NSCLC patients with CNS metastases. **(A, B)** The actionable mutation detection rates of different groups; **(A)** CNSM group: distant metastases only from the CNS group; **(B)** CNSM+ group: distant metastases other than CNS metastases group. **(C)** Actionable mutations detected in different samples from treated NSCLC patients with CNS metastases. CNSM group, patients had distant metastases only from the CNS, CNSM+ group, patients with other organ involvement and distant metastases other than CNS metastases.

To further analyze the detective capability of actionable mutations of CSF samples in a real-world setting, 1,156 treated samples from NSCLC patients with CNS metastases were retrospectively analyzed. Thus, we compared all actionable mutations and *EGFR*, *ALK*, *ROS1*, *BRAF*, *KRAS*, *MET*, *RET*, and *ERBB2* alterations in patients in the CNSM (distant metastases only from the CNS) and CNSM+ (distant metastases other than CNS metastases) groups. The actionable mutation detection rates of the different groups are shown in **Figures 6A, B**, and the actionable mutation detection numbers of different groups are shown in **Figure 6C**.

Compared to tumor tissue, the detection rates of actionable *EGFR* in CSF were significantly higher than those in tissue samples in both the CNSM and CNSM+ groups (all p <0.001) (**Figures 6A, B**). CSF and tumor tissue had similar sensitivity in detecting all actionable mutations in both the CNSM and CNSM+ groups (89.58% vs. 94.77% for the CNSM group, P = 0.2008; 95.65% vs. 97.38% for the CNSM+ group, P = 0.4848) (**Figures 6A, B**). The detection rates of actionable *EGFR* and all actionable mutations in CSF were significantly higher than those in plasma samples in both the CNSM and CNSM+ groups (all p <0.0001) (**Figures 6A, B**).

For CSF, the *EGFR* and all actionable mutation detection rates were not statistically different between the CNSM and CNSM+ groups (p = 0.1977 for the CNSM group; p = 0.1001 for the CNSM+ group) (**Figures 6A, B**). For plasma, detection rates of actionable *EGFR* and all actionable mutations in the CNSM+ group were significantly higher than in the CNSM group (all p <0.0001) (**Figures 6A, B**). CSF showed a potential capability to detect actionable mutations.

### Concordance of Paired CSF and Plasma Samples

**Figure 7:**
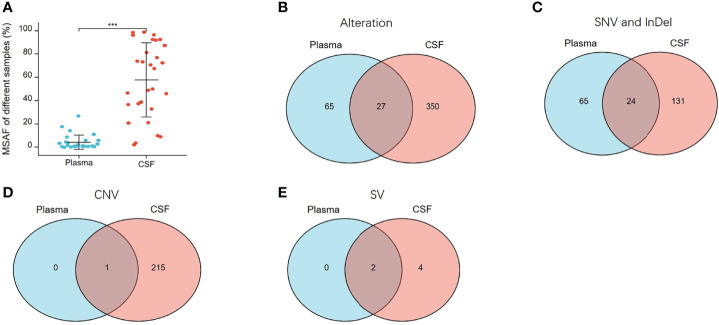
MSAF and concordance of 32 paired CSF and plasma samples. **(A)** MSAF of 32 paired CSF and plasma samples from 28 treated-patients. **(B)** The concordance of CSF and plasma samples for detection of all alterations. **(C)** The concordance of CSF and plasma samples for detection of SNV or InDel. **(D)** The concordance of CSF and plasma samples for detection of CNV. **(E)** The concordance of CSF and plasma samples for detection of SV. ***p < 0.001.

To verify the results described above, a concordance analysis was performed on 28 treated patients with 32 pairs of paired CSF and plasma samples, which were simultaneously tested for 1,021 cancer-relevant genes. In all 32 paired samples, the detection rate of cfDNA in CSF and plasma was the same (90.62%, 29/32). The MSAF of 32 CSF cfDNAs was compared to that of plasma cfDNA from 28 patients. The MSAF of the CSF was significantly higher than that of the matched plasma cfDNA (p <0.001) (**Figure 7A**).

In the 32 paired plasma and CSF samples, 442 alterations were detected, 377 and 92 of which were detected in CSF and plasma, respectively. As shown in **Figure 7B**, 27 alterations were detectable in both plasma and CSF, 65 were undetectable in CSF, and 350 were undetectable in plasma. The same alterations were 10.91% (27/442). For SNV or InDel, 220 mutations were detected, of which 155 were detected in CSFs and 89 in plasma. The same mutations were 10.91% (24/220) (**Figure 7C**). For CNV, 216 CNV alterations were detected, of which 216 were detected in CSF and only one in plasma (**Figure 7D**), and for SV, six SV alterations were detected, of which six were detected in CSF and two in plasma (**Figure 7E**). From multiple comparisons, it is evident that CSF can better detect alterations than plasma, especially CNV and SV alterations.

### CSF for TKI-Resistance Mechanisms Exploring

In this retrospective cohort, seventy-seven NSCLC patients with CNS metastases who had previously received targeted treatment with EGFR- or ALK-, or ROS1-tyrosine kinase inhibitors (TKIs) were tested for NGS using CSF samples. We successfully tested 100 CSF samples obtained from 77 patients, of which 22 patients were resistant to first or second-generation EGFR-TKIs, 49 were resistant to osimertinib or almonertinib, and 6 were resistant to ALK- or ROS1-TKIs (**Table 2**).

**Table 2:** Known resistant mutations detected in CSF samples from NSCLC patients with CNS metastases who had previously received targeted treatment.

	1st/2nd-generation EGFR-TKIs (n = 22, sample = 26)	Osimertinib/almonertinib (n = 49, sample = 67)	ALK- or ROS1-TKIs (n = 6, sample = 7)
EGFR T790M	/	–	–
C797X, L792X G724S, L718Q	–	8	–
ALK/ROS1 SNV	–	–	1
PI3K-AKT-mTOR signaling-related genomic alteration	1	7	/
KRAS mutation	/	1	/
ERBB2 amplification	/	4	/
MET amplification	3	9	/
cell cycle gene alterations	5	6	–
*EGFR* amplification	9	34	–
*TP53* exon8 mutation	6	7	–
*TP53* mutation	–	–	2
without sensitizing mutations	1	3	2
Total	25	79	5

While exploring mechanisms of TKI-resistance, EGFR-TKI sensitizing mutations were undetected in CSF cfDNA in 4.30% (4/93) of patients with EGFR-TKI resistance. Fifty-six patients (78.87%, 56/71) harbored concurrent alterations that might limit the efficacy of EGFR-TKIs, namely, *EGFR* resistance mutation, activation of bypass signaling pathways, *EGFR* amplification, *TP53* exon8 mutation, PI3K-AKT-mTOR gene alterations, and cell cycle gene alterations. In total, known EGFR-TKIs resistance mechanisms, such as PI3K-AKT-mTOR signaling-related genomic alteration, *MET* amplification, and absence of sensitizing mutations were detected in 1, 3, and 1 patient(s), respectively, who were resistant to first- or second-generation EGFR-TKIs. *EGFR* C797S/L792X/G724S/L718Q, PI3K-AKT-mTOR signaling-related genomic alteration, *KRAS* mutation, *ERBB2* amplification, *MET* amplification, and absence of sensitizing mutations were detected in 8, 7, 1, 4, 9, and 3 patients, respectively, who were resistant to osimertinib or almonertinib. Cell cycle gene alterations, *EGFR* amplification, and *TP53* exon8 mutations were identified in 11, 43, and 13 patients with EGFR-TKI resistance, respectively. However, determining whether it would result in EGFR-TKI resistance remains controversial. Co-occurrence of resistance mechanisms was observed in 21 patients, including one patient without EGFR-TKIs sensitizing mutations.

Four patients with ALK- or ROS1-TKI resistance were identified as having *ALK* or *ROS1* fusions. Known ALK-TK resistance mechanisms, such as *ALK* G1269A and absent sensitizing mutations, were detected in one and two of five patients with ALK-TKIs resistance. *TP53* mutations that may limit the efficacy of ALK-TKIs were identified in two patients with ALK-TKI resistance.

This confirmed that liquid biopsy using CSF showed high potential in exploring underlying resistance mechanisms in NSCLC patients with CNS metastases.

## Discussion

NSCLC patients with CNS metastases usually have a poor prognosis and limited treatment options. However, following the development of cancer genomics and more effective targeted therapies, new treatments are emerging (4–7, 9). Genotyping can provide genomic information and evolutionary patterns of CNS metastases in NSCLC patients, which may be key in using targeted therapeutic strategies (14, 29). However, because CNS tissue collection is difficult and invasive, and plasma insensitivity owing to its inability to penetrate the blood–brain barrier develops, it is clinically challenging to select a suitable sample for genetic testing and genotyping in clinical practice (15, 21).

Among NSCLC patients with CNS metastasis, ctDNA in CSF, which circulates throughout the CNS, can reveal genomic alterations in intracranial lesions (30–32). Previous studies indicated that CSF ctDNA was more exact and complete than plasma ctDNA and could thus be an optimal source of liquid biopsy for genotyping to guide therapy and predict prognosis (21–24). CSF genetic alterations have been associated with the survival of advanced lung adenocarcinoma patients with CNS metastases (24). However, only a limited number of NSCLC patients with CNS metastases were included in these studies; furthermore,the potential use of CSF in real-world settings is yet to be examined.

To date, this real-world study recruited the largest cohort of NSCLC patients with CNS metastases who had CSF or other samples available for NGS testing. All CSF samples in this study were tested using the CSF supernatant, as it was reported that more mutations could be detected in cfDNA from the CSF supernatant than in paired CSF cells because CSF cfDNA was less affected by non-tumor cell components (30, 33). Comparisons between groups divided according to treatment history and sites of metastasis revealed the choice of specimen type for genetic profiling among these NSCLC patients in the real world. For NSCLC patients with CNS metastases, tissue and plasma samples were the most frequently examined in treatment-naive patients and treated patients, respectively. It is worth mentioning that only 11.06% (46/416) of all tissue samples were intracranial lesions, and there was heterogeneity between intracranial and extra-cranial lesions (14); therefore, extra-cranial lesions may not be the optimal sample for NSCLC patients with CNS metastases. CSF was the choice for genetic profiling in all groups, and treated patients who had distant metastases only from the CNS (10.96%) had the largest proportion of CSF samples.

This study also investigated the genetic alterations in CSF samples from NSCLC patients. Alteration rates (including all alterations, SNV or Indels, and CNV), especially CNV alterations, in the CSF samples were significantly higher than those in plasma (all p <0.001), which corresponded to the findings of other reports (16). The MSAFs of the CSF samples were significantly higher than those of plasma and tumor tissues (all p <0.001). We speculated that the lower MSAF in plasma may account for the inferior detection efficacy of plasma compared to CSF and tissues. A comparison of the genetic profiles in different treated-samples indicated that *EGFR* and *TP53* were the two most frequently mutated genes in all sample types, which were the same as previously reported (16). These real-world data suggest that cfDNA isolated from CSF can effectively provide important genomic information about an individual tumor and also that CSF can be used as a substitute in the absence of intracranial tumor tissue.

For driver gene alterations, the detection rate of *EGFR*, *ALK*, *ROS1*, *BRAF*, *KRAS*, *RET*, *MET*, and *ERBB2* alterations in the tissues was consistent with previous reports (34). Remarkably, the detection rates of all actionable mutations and *EGFR* in CSF were higher than those in plasma samples; moreover, *EGFR* in CSF was higher than that in tissue samples from treated patients, regardless of distant metastases other than CNS metastases (all p <0.0001). Thus, for treated NSCLC patients with CNS metastases, CSF outperformed plasma in detecting actionable mutations. For concordance between paired CSF and plasma samples, the MSAF of the CSF was significantly higher than that of the matched plasma cfDNA (p <0.001). From multiple comparisons, it was seen that CSF is better than plasma in detecting alterations, especially CNV and SV alterations.

This study investigated the performance of CSF in detecting resistance mutations among treated patients. CSF cfDNA for identifying mutations can show why the efficacy of EGFR-TKIs was limited in 56 patients (78.87%, 56/71). A recent study evaluated the role of CSF-NGS in osimertinib-treated *EGFR*-mutated NSCLC and found that the detection rate of *EGFR* mutations using CSF genotyping was 97.1%, compared to 95.5% in our study (35). It also showed that CSF might reveal resistance mechanisms such as C797S mutation, MET dysregulation, and cell cycle pathway alterations implicated in osimertinib failure, which our study also confirmed (35). A previous study confirmed that identifying resistant ALK secondary mutations is essential in ALK fusion-positive NSCLC patients progressing after ALK-TKI therapy, as it can influence sensitivity to subsequent ALK-TKIs (36). In this study, patients with ALK-TKI resistance were identified with ALK secondary mutations using CSF cfDNA, and patients with ALK- or ROS1-TKI resistance were identified as having an ALK or ROS1 fusion and known ALK-TKI resistance mechanisms. We demonstrated that CSF cfDNA was more informative in identifying secondary mutations among drug-resistant patients and initial sensitive mutations than plasma, as previously reported. CSF cfDNA could be used for intracranial biopsy to test for acquired resistance in NSCLC patients with CNS metastases.

Precision medicine has led to improvements in the prognosis of patients with advanced NSCLC. Adoption of NGS is time-saving and cost-efficient (37). For NSCLC patients with CNS metastases, the selection of the appropriate sample type for NGS to save time and reduce the overall cost of testing is important. Real-world data demonstrates that analysis of cfDNA isolated from CSF may provide important genomic information for NSCLC patients with CNS metastases. Overall, more treated patients choose CSF for genetic testing than treatment-naive patients do. However, fewer patients choose CSF as a test sample than plasma or tissue in the real world, possibly because it is more traumatic and more difficult to perform a lumbar puncture than to obtain CSF from blood. Therefore, it is important to select NSCLC patients with CNS metastases who are clinically suitable for CSF testing. Meanwhile, a previous study reported that clinical factors such as the diameter of the largest intracranial lesion and the minimum lesion–ventricle distance for all intracranial lesions had significant influence on the detection of CSF (38). In clinical practice, specific clinical manifestations of patients can be combined to select suitable specimens. However, plasma is the preferred choice for molecular profiling in all groups in the real-world, especially in treated patients. Meanwhile,the detection rate was unsatisfactory, especially in patients who had distant metastases only from the CNS, and in the detection of CNV alteration. Moreover, the detection rate of genomic alterations has been reported to be lower in the plasma ctDNA of patients with isolated CNS disease, and complementary tests such as CSF cfDNA may be useful for these patients (39). Therefore, for patients with isolated CNS metastases and those who have retested CNV mutations, other samples such as CSF are recommended for real-world genetic testing.

This study has some limitations, which include its retrospective snapshot study design, without consideration of additional clinical factors and therapeutic efficacy. Hence, we could not precisely determine optimal specimens for patients in different clusters. The number of treatment-naive patients included in this study was small, and the role of CSF in this subset of patients is limited. Furthermore, the paired CSF and plasma samples were small, and no tissue samples were included. Therefore, we could not investigate concordance between tissue, plasma, and CSF at the same time point. The clinical implications of CSF genotyping on treatment outcomes were not analyzed in this study.

## Conclusions

This large-scale, real-world study confirmed that liquid biopsy using CSF showed high potential for identifying actionable mutations and exploring the underlying resistance mechanisms in NSCLC patients with CNS metastases. CSF can be used as a liquid biopsy source to facilitate the broad exploration of potential resistance mechanisms in clinical practice.

## Data Availability Statement

The original contributions presented in the study are publicly available. This data can be found in the GVM in National Genomics Data Center, China National Center for Bioinformation / Beijing Institute of Genomics, Chinese Academy of Sciences, under accession number GVM000345, https://ngdc.cncb.ac.cn/gvm/getProjectDetail?project=GVM000345.

## Ethics Statement

The studies involving human participants were reviewed and approved by the Institutional Review Board of Shaanxi Provincial People’s Hospital. The patients/participants provided their written informed consent to participate in this study. Written informed consent was obtained from the individual(s) for the publication of any potentially identifiable images or data included in this article.

## Author Contributions

FS, JB, NL, WG, and ZF contributed to conception and design of the study. JB, JZ, WG, GD, MZ, and QH organized the database. KW and MY performed the statistical analysis. KW and RC wrote the first draft of the manuscript. KJ, XW, YM, and GD wrote sections of the manuscript. All authors listed have made a substantial, direct, and intellectual contribution to the work and approved it for publication.

## Funding

This work was supported by the National Natural Science Foundation of China (82072583), the Beijing Municipal Administration of Hospitals Incubating Program (PX2020044), and the Beijing Municipal Science & Technology Commission (No. Z171100000417029).

## Conflict of Interest

KW, MY, and RC are current employees of Geneplus-Beijing.

The remaining authors declare that the research was conducted in the absence of any commercial or financial relationships that could be construed as a potential conflict of interest.

## Publisher’s Note

All claims expressed in this article are solely those of the authors and do not necessarily represent those of their affiliated organizations, or those of the publisher, the editors and the reviewers. Any product that may be evaluated in this article, or claim that may be made by its manufacturer, is not guaranteed or endorsed by the publisher.

## Abbreviations

CSF, cerebrospinal fluid; CNS, central nervous system; NSCLC, non-small cell lung cancer; NGS, next-generation sequencing; *EGFR*, epidermal growth factor receptor; *ALK*, anaplastic lymphoma kinase; *ROS1*, ROS proto-oncogene 1; ctDNA, circulating tumor DNA; IASLC, International Association for the Study of Lung Cancer; BM, brain metastases; LM, leptomeningeal metastases; WBC, white blood cell; FFPE, formalin-fixed paraffin-embedded; cfDNA, circulating cell-free DNA; SNV, somatic single nucleotide variation; Indel, insertion or deletion of small fragment; CNV, copy number variants; SV, structural variants; MSAF, maximal somatic allele frequency; TKI, tyrosine kinase inhibitor.
